# ABO and Rh Blood Type Relationship in Parents with more than One Disabled Child 

**Published:** 2015-12-10

**Authors:** M Mehrmohammadi

**Affiliations:** Department of Psychology, Behbahan Branch, Islamic Azad University, Behbahan, Iran

**Keywords:** Blood Type, Intellectual disability, Mental Retardation, Rhesus Blood

## Abstract

**Background:**

Parental blood variables are one of the most important medical-biological causes of intellectual and physical-movement disabilities. The purpose of this study was to identify the relationship between parents’ blood variables (ABO and Rh blood type) and their relationship with frequency of intellectual and physical-movement disabilities in Isfahan province.

**Materials and Methods:**

This was a descriptive-analytical study and 494 samples were selected from mothers with more than one disabled child and mothers with normal child using simple and multistage random methods. The data collection was done through questionnaire. Based on Kuder-Richardson Formula 20 (KR-20), the reliability of questionnaire was 0.88. The statistical model in this study was a hierarchical log-linear method.

**Results:**

There was a significant relationship between mother’s Rh blood and having disabled child (P=0.002). However no significant relationship between having disabled children and the following variables was found: the father’s Rh blood (p=0.2), father and mother’s Rh blood together (P=0.5), father blood type (P=0.56), mother blood type (P=0.42), and mother and father blood types together (P=0.7).

**Conclusion:**

Maternal and fetal blood incompatibility (motherwith negative Rh blood and fetus with positive Rh blood) increased the likelihood of being born with disabilities.

## Introduction

Studies showed that some parts of Iran allocated the highest average of intellectual and physical-movement disabilities to themselves compared to international averages (1).at the current time, there are 222,881 families in province of Isfahan who have one or more disabled children (2). The profusion number of disabled individuals is one of the most serious problems in Isfahan.

American Association on Intellectual and Developmental Disabilities (2002) categorizes the effective factors in causing disability into 4 groups including medical-biological factors, social factors, behavioral factors, and educational factors. 

Blood factors are one of the most important medical-biological causes in etiology of intellectual and physical-movement disabilities (3). Blood types are distinguished by two features: blood types and Rh status. If the male is Rh positive and the female is Rh negative, the baby will most probably be Rh positive (4). Rh blood incompatibility is the major cause of hemolytic disease (5). Rh blood incompatibility can lead to hyperbilirubinemia which if not treated on time can cause chronic stress. The latter damages the central nervous system andfollowed by outcomes such as miscarriage, stillbirth, cerebral palsy, paralysis of limbs, and intellectual disability in the infant (6,7). Beside Rh, the other factor associated with pregnancy complications is the ABO blood system. Today, it is known that some blood antigens increase the individual’s susceptibility to certain diseases and its impositions (8). Abnormal bleeding during pregnancy and c-section rates are higher in women with blood type A than in other blood types. Still birth and pregnancy edema are less frequent in blood type AB and birth weight < 2500 grams and death in the first week of birth in blood type O are less common than in other blood types (9). Bipolar disorder type 1 has been reported more frequently in blood type O (10). In addition, there has been a significant relationship between the parents’ blood type and the birth of children with cerebral palsy (11). The blood type of father has been significantly related to the occurrence of Down syndrome. In other words, the incidence of Down syndrome has been more common when fathers had blood type O whereas its incidence has been less common when fathers had blood type B positive. Moreover, among 64 different combinations of parents’ blood compositions, the blood composition of mother O negative plus father O positive, and mother A positive plus father A negative increased the risk of Down syndrome incidence (12). Blood type O positive and negative and also a positive were less common in mothers with more than one disabled child than in the ones with normal children. Furthermore, blood type B positive was more common in fathers with more than one disabled child than in the ones with normal children. In general, the purpose of this study was to identify the association between parents’ ABO and Rh blood type with intellectual and physical-movement disabilities in newborns of Isfahan province.

## Material and Methods:

This was a descriptive-analytical study. The current study aimed to answer two major questions as follows:

1. Is there any relationship between father and mother’s Rh blood with having more than one disabled child?

2. Is there any relationship between mother and father’s blood type ABO with having more than one disabled child?

The study population included parents with more than one disabled child intellectual or physical-movement disabilities, in Isfahan province. The intellectual disability in this study was defined as mental retardation. Physical disability included cerebral palsy, paralysis of limbs, congenital hip dislocation, dystrophy, etc. Parents with more than one disabled child, either intellectual or physical-movement disabilities, were placed in the case group whereas parents with normal children were categorized as control group. The parents’ selection was done after approval of ethics committees of the Health Welfare Institution of Isfahan province and Isfahan Health Networks districts 1 and 2 and based on the lists provided by these centers. The parents with disabled children were selected using multistage random sampling method from Isfahan province in 2011. The case group was selected from mothers who had at least two disabled children (either intellectual or physical-movement disabilities). The selected parents with disabled children were matched with the parents with normal children in terms of the number of children and geographic region. In the current study, 494 samples were studied of whom247 were mothers with more than one disabled child and 247 were mothers with normal children. The sample size was justifiable according to the statistical population model. The selected samples were completely informed orally and by written documents about the study process and they agreed to take part in the study. The data collection tool was a questionnaire made by the researcher. The questionnaire was approved by seven experts in this field and met the content validity ratio. Moreover, based on Kuder-Richardson Formula 20 (KR-20), the reliability of questionnaire was 0.88. Noting that the independent variables (ABO and Rh blood types) and dependent variable (disabled child birth) were categorized in this study, the hierarchical log linear statistical model, which is the most appropriate model for discrete multivariate data analysis, was used for data analysis. The main effects, two-way and three-way links were examined by the same method. The data were analyzed using SPSS version 16. The data was collected through interview.

## Results

In this study, Frequency distribution of mother and fathers’ RH blood in parents with disabled and normal children is mention in table 1. Models related to effects of multivariate and higher interactions and coefficients of the partial match between parents’ Rh blood and the related group are mention in table 2 and 3 respectively. The result showed that the most common Rh compositions in both groups was observed when both parents were Rh positive and the least common was observed when they were both Rh negative. The descriptive statistics showed that the combination of mother with Rh negative and father with Rh positive (Rh blood incompatibility) was significantly more common in parents with disabled children than in the ones with normal children. Furthermore, there was a significant relationship between mother’s Rh blood and having disabled children. The findings did not demonstrate any significant relationship between father’s Rh blood and having disabled children. The frequency distribution of blood type in parents with disabled children and normal children are demonstrated in Table 4. the following blood components were observed more frequently in parents with childrenthan in the ones with normal children: mother A and father AB, mother B and father A, mother and father both B, mother AB and father A, mother AB and father B, and mother O and father AB. The three-way link among having disabled children, mother’s blood type and father’s blood type was not statically significant (Table 5). The findings related to the question (Table 6) showed that from the two-way interactions, only mother and father’s blood type were interacted significantly.

**Table I:** Frequency distribution of mother and fathers’ RH blood in parents with disabled and normal children

Groups	Mother Rh Blood	Father Rh Blood	Observed Frequency Percentage	Expected Frequency Percentage
Parents with disabled children	Positive Negative	Positive Negative Positive negative	115 30.1% 20 5.2% 29 7.6% 7 1.8%	126.8 33.2% 19.1 5% 21.78 5.7% 3.28 0.9%
Parents with normal children	Positive Negative	Positive Negative Positive negative	172 45% 19 5% 16 4.2% 4 1%	156.5 41% 23.56 7% 26.8 7% 4.04 1.1%

as follows: mother and father both Rh positive 45%, mother Rh positive and father Rh negative 5%, mother Rh negative and father Rh positive 4.2%, and mother and father both Rh negative 1% .

**Table II:** Models related to effects of multivariate and higher interactions (corresponding to the first question)

K	Degree of freedom (DOF)	Ratio G^2^	Likelihood Significance	Pearson *x*^2^	Significant level
Multivariate and higher interaction effects	1 7 2 4 3 1	462.5 14.11 0.38	0.000 0.007 0.5	554.7 14.5 0.39	0.000 0.000 0.5
Multivariate interaction effects	1 3 2 3 3 1	448.4 13.7 0.38	0.000 0.003 0.5	540.2 14.1 0.39	0.000 0.000 0.5

**Table III:** Coefficients of the partial match between parents’ Rh blood and the related group

Effects	G^2^	Degree of freedom (DOF)	Significant level
Having disabled children and mother’s Rh blood	9.5	1	0.002
Having disabled children and father’s Rh blood	1.37	1	0.2
Mother and father’s Rh Blood	1.64	1	0.2
Parents with normal children	4.19	1	0.04
Mother’s Rh blood	211.16	1	0.000
Father’s Rh blood	233.079	1	0.000

**Table IV:** distribution of mother and fathers’ blood type in parents with disabled and normal children

Group	Mothers’ blood type	Father’s blood type	Observed Frequency Percentage	Expected Frequency Percentage
Parents with disabled children	A	A B AB O	28 7.3% 7 1.8% 8 2.1% 11 2.9%	20.081 5.3% 10.997 2.9% 7.172 1.9% 22.631 5.9%
	B	A B AB O	7 1.8% 11 2.9% 5 1.3% 9 2.4%	9.320 2.4% 5.094 1.3% 3.322 0.9% 10.483 2.7%
	AB	A B AB O	5 1.3% 4 1% 4 1% 5 1.3%	5.611 1.5% 3.073 0.8% 2.004 0.5% 6.323 1.7%
	O	A B AB O	17 4.5% 12 3.1% 5 1.3% 33 8.6%	21.410 5.6% 11.724 3.1% 7.646 2% 24.128 6.3%
Parents with normal children	A	A B AB O	41 10.7% 11 2.9% 6 1.6% 24 6.3%	24.778 6.5% 13.569 3.6% 8.894 2.3% 27.924 7.3%
	B	A B AB O	6 1.6% 9 2.4% 5 1.3% 11 2.9%	11.478 3% 6.286 1.6% 4.099 1.1% 12.936 3.4%
	AB	A B AB O	4 1% 1 0.3% 9 2.4% 6 1.6%	6.923 1.8% 3.791 1% 2.473 0.6% 7.802 2%
	O	A B AB O	18 4.7% 14 3.7% 3 0.8% 43 11.3%	6.418 6.9% 14.476 3.8% 9.435 2.5% 29.772 7.8%

**Table V:** Models related to effects of multivariate and higher interactions (corresponding to the second question)

K	Degree of freedom (DOF)	Ratio G^2^	Likelihood Significant	Pearson *x*^2^	Significant level
Multivariate and higher interactions effects	1 31 2 24 3 9	239.5 70.6 6.4	0.000 0.000 0.7	297.5 77.5 6.2	0.000 0.000 0.7
Multivariate interactions Effects	1 7 2 15 3 9	448.4 13.7 0.38	0.000 0.000 0.7	219.9 71.3 6.2	0.000 0.000 0.7

**Table VI:** Coefficients of the partial match between parents’ blood type

Effects	G^2^	Degree of freedom (DOF)	Significant level
Parents with disabled children and mother’s blood type	2.82	3	0.42
Parents with disabled children and father’s blood type	2.02	3	0.56
Mother and father’s blood types	59.9	9	0.000
Parents with normal children	4.2	1	0.04
Mother’s blood type	94.8	3	0.000
Father’s blood type	69.9	3	0.000

## Discussion

In relation to the first question, the current study showed that there was a significant relationship between mother’s Rh blood and having disabled children. In other words, mother’s Rh blood incompatibility (mother with Rh negative and fetus with Rh positive) will increase the likelihood of children born with disabilities (intellectual and/or physical-movement disability). The same blood incompatibility causes the outbreak of hemolytic disease which if not treated on time leads to chronic stress followed by damages to the central nervous system. The damages to the central nervous system will increase the chance of miscarriage, stillbirth, cerebral palsy, paralysis of limbs, and intellectual disability (6). In confirmation of the current findings, Sediqi and Majlesi (2002), Rezaie and colleagues (2008), Afrouz and colleagues (2009) studies showed that mother’s blood incompatibility would increase the likelihood of the birth of disabled child (11-13). In relation to second question, the findings showed a significant relationship between mother and father’s blood types. The most frequent blood type in both groups was O. In accordance with the findings of the current study, Mahmoudi’s studies (2005) and Afrouz and colleagues (2009) no significant relationship between father’s blood type and having disabled children, mother and father’s blood types, father’s blood type and mother’s blood type and having disabled children were observed (12,14). In line with the discretional data collected, Pour-Jafari and Colleagues’ studies (2003), the most frequent blood type in both groups was O , A,and B (15), respectively. Moreover, Soleymani and colleagues’ studies indicated a significant relationship between blood types of parents with more than one disabled child (16).

## Notes

**Conclusion:** Maternal and fetal blood incompatibility (motherwith negative Rh blood and fetus with positive Rh blood) increased the likelihood of being born with disabilities.
